# Precise and simultaneous quantification of mitochondrial DNA heteroplasmy and copy number by digital PCR

**DOI:** 10.1016/j.jbc.2022.102574

**Published:** 2022-10-06

**Authors:** Wendy K. Shoop, Cassandra L. Gorsuch, Sandra R. Bacman, Carlos T. Moraes

**Affiliations:** 1 Precision BioSciences, Durham, North Carolina, USA; 2 University of Miami Miller School of Medicine, Miami, Florida, USA

**Keywords:** mitochondrial DNA, heteroplasmy, mtDNA copy number, digital PCR, MELAS, mitochondrial genetics, Ct, cycle threshold, dPCR, digital PCR, mtDNA, mitochondrial DNA, nDNA, nuclear DNA, qPCR, quantitative PCR, RFLP, restriction fragment-length polymorphism

## Abstract

Mitochondrial DNA (mtDNA) is present in multiple copies and phenotypic consequences of mtDNA mutations depend on the mutant load surpassing a specific threshold. Additionally, changes in mtDNA copy number can impact mitochondrial ATP production, resulting in disease. Therefore, the precise determination of mtDNA heteroplasmy and copy number is crucial to the study of mitochondrial diseases. However, current methods can be imprecise, and quantifying small changes in either heteroplasmy or copy number is challenging. We developed a new approach to measure mtDNA heteroplasmy using a single digital PCR (dPCR) probe. This method is based on the observation that fluorescent-labeled probes in dPCR exhibit different intensities depending on the presence of a single nucleotide change in the sequence bound by the probe. This finding allowed us to precisely and simultaneously determine mtDNA copy number and heteroplasmy levels using duplex dPCR. We tested this approach in two different models (human and mouse), which proved faster and more internally controlled when compared to other published methods routinely used in the mitochondrial genetics field. We believe this approach could be broadly applicable to the detection and quantification of other mixed genetic variations.

Mitochondria, the ATP-producing organelles present in nearly all eukaryotic cells, contain their own multi-copy genome that is susceptible to mutation (1). Defects within the mitochondrial DNA (mtDNA) can impair key mitochondrial activities, such as ATP production *via* oxidative phosphorylation, resulting in disease (2, 3). Pathogenic mtDNA mutations are commonly heteroplasmic, where both WT and mutant genomes coexist in the same cell (1, 4). It is not until the level of mutant mtDNA exceeds a particular disease threshold that clinical symptoms manifest. This threshold varies based on the specific mutation and target tissue but is typically between 60 to 95% (5). In addition to heteroplasmic mtDNA mutations, mtDNA depletion syndromes that manifest with severe mtDNA reduction can also lead to defects in mitochondrial ATP production, resulting in disease (6). Therefore, the accurate quantification of the heteroplasmy level and mtDNA copy number in a given tissue or cell population is vital to understand disease severity and progression. The most widely used methods to quantify mtDNA heteroplasmy are PCR/restriction fragment-length polymorphism (RFLP), which relies on the removal or addition of a unique restriction enzyme cut site by the mutation, or Sanger sequencing. PCR/RFLP has limitations due to heteroduplex formation during PCR (7), while Sanger sequencing can detect heteroplasmy as low as 15% but peak height calling at polymorphic sites can be imprecise (8). Although quantitative PCR (qPCR) has also been used to quantify mtDNA heteroplasmy (9), it requires one assay corresponding to each haplotype (mutant and WT). qPCR is also the most commonly utilized method to quantify mtDNA copy number (10), although changes smaller than 2-fold cannot accurately be quantified (11). We sought to streamline and improve the precision of mtDNA heteroplasmy and copy number quantifications by harnessing the sensitivity, quantitative, and high-throughput capabilities of digital PCR (dPCR).

dPCR has emerged as the gold standard for nucleic acid quantification, particularly for low-abundance targets. dPCR is similar to qPCR in that both can utilize hydrolysis probes specific to a target DNA sequence. However, dPCR does not require a standard curve and instead relies on massive sample partitioning and Poisson statistics to absolutely quantify the amount of target DNA within a sample. In dPCR, the bulk PCR reaction is partitioned, resulting in a random distribution of target and background DNA amongst the partitions (or droplets in the case of droplet dPCR). PCR amplification then occurs within each individual partition. Any partitions that contain the target DNA sequence will accumulate the fluorescent signal associated with the respective probe. Following amplification, each partition is analyzed for fluorescence and categorized as either positive or negative. Partitions which contain at least one copy of the target DNA sequence are considered positive because they exhibit increased fluorescence relative to the negative partitions, which contain no copies of the target DNA sequence. By fitting the fraction of positive partitions within the analyzed sample to a Poisson algorithm, the starting concentration of target DNA within the sample can be absolutely quantified.

The high sensitivity of dPCR makes it an ideal method for the detection of single nucleotide polymorphisms (SNPs), such as heteroplasmic mtDNA mutations. Current dPCR methods used to detect and quantify the frequency of a given SNP within a DNA sample utilize two probes, each with a different fluorophore, to distinguish the different alleles (12) (https://www.bio-rad.com/webroot/web/pdf/lsr/literature/Bulletin_6628.pdf). Obtaining the absolute quantification of both alleles then allows the relative frequency of each to be calculated. In this study, we demonstrate the ability of dPCR to quantify mtDNA heteroplasmy using a single probe.

dPCR by definition is binary: partitions are either positive or they are negative. However, partitions that are positive can vary in the amplitude of fluorescent signal they emit because the fluorescent signal found in each partition is proportional to the amount of amplified product within the partition. We discovered that by using a probe designed to specifically detect the mutant haplotype for a particular mtDNA point mutation, we were still able to amplify the WT mtDNA haplotype—just at a lower efficiency. This lower efficiency resulted in a lower intensity, but still positive, population of partitions. The observed difference in fluorescent signal between the two haplotypes resulted in distinct partition populations which could be clearly separated and analyzed. Fig. 1 outlines this approach. By utilizing a single primer/probe set to quantify mtDNA heteroplasmy, we found we were able to duplex with a multi-copy nuclear DNA (nDNA) reference assay to simultaneously quantify mtDNA heteroplasmy and mtDNA copy number, which to our knowledge has not previously been reported.

**Figure 1:**
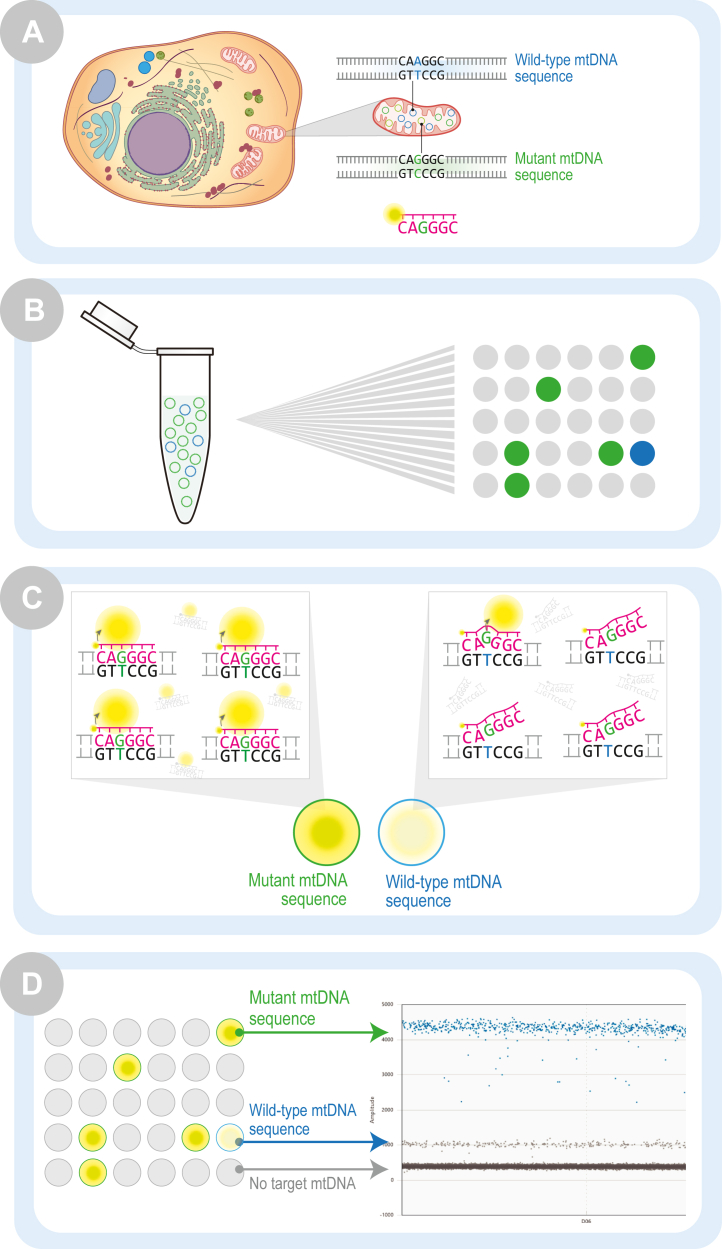
**Quantification of mtDNA heteroplasmy using a single dPCR probe**. *A*, mtDNA mutations are commonly heteroplasmic, with both mutant and WT mtDNA present in the same cell. The abundance of each haplotype can be quantified using a fluorescent-labeled dPCR probe specific to one of the haplotypes, in this case the mutant mtDNA sequence. *B*, a bulk reaction mixture containing both mtDNA sequences is fractionated into partitions. Some partitions will contain WT mtDNA, some will contain mutant mtDNA, and some will contain no target DNA. PCR is carried out within each partition. *C*, when the mutant mtDNA sequence is present, efficient probe binding results in abundant fluorescent signal. When the WT mtDNA sequence is present, less efficient probe binding results in diminished, yet still detectable fluorescent signal. *D*, each partition is analyzed as either positive or negative for the fluorescent signal. Partitions are graphically distributed based on the abundance of fluorescent signal present in the partition where more fluorescent signal produces a higher amplitude partition population. mtDNA, mitochondrial DNA; dPCR, digital PCR.

## Results

### A single dPCR probe can discriminate mtDNA heteroplasmy

We set out to quantify mtDNA heteroplasmy at position 3243 of the human mitochondrial genome. The WT adenine at position 3243 (tRNA Leu (UUR) gene) is frequently mutated to a guanine (m.3243A > G), and this point mutation is implicated in >80% of cases of mitochondrial encephalomyopathy, lactic acidosis, and stroke-like episodes (13). Biosearch Technologies utilizes a proprietary duplex-stabilizing chemistry to generate DNA probes with elevated melting temperature and enhanced target specificity, called BHQplus probes. Two BHQplus probes were designed to specifically bind to either the mutant 3243G or WT 3243A mtDNA sequence. Using the same primer pair with each probe, these assays are henceforth referred to as either the mutant mtDNA assay or WT mtDNA assay. Additionally, an mtDNA assay utilizing a different fluorophore was designed in the MT-ND2 gene to serve as a reference mtDNA assay.

**Figure 2:**
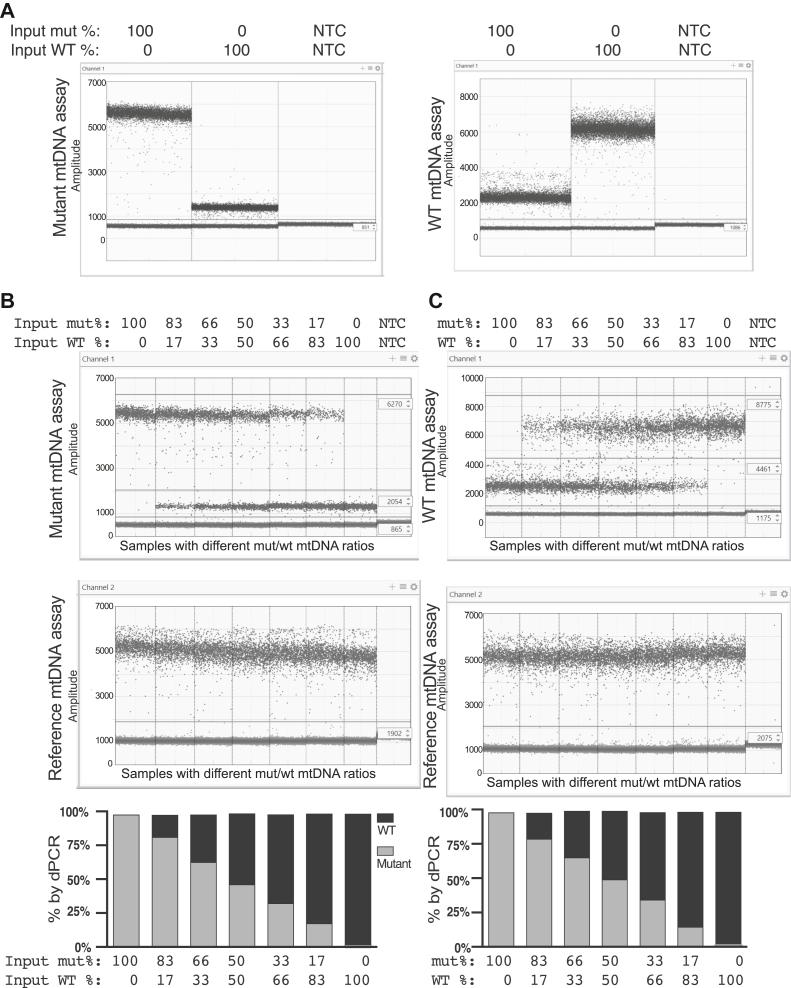
**A dPCR probe can discriminate a single nucleotide change.***A*, simplex droplet dPCR plots for the mutant mtDNA assay (*left*) and WT mtDNA assay (*right*) using synthetic DNA input. Both assays utilized FAM-labeled probes. Individual wells are separated by vertical lines, and the synthetic DNA input for each well is noted above the droplet plot. Approximately, 20,000 partitions (droplets) are shown within each well. The fluorescent signal of each droplet is graphically displayed according to the intensity of the signal, with stronger signal appearing at a higher amplitude. The *solid horizontal line* indicates the threshold between negative and positive droplets. *B*, duplex droplet dPCR plots for the mutant mtDNA assay (*top panel*) and reference mtDNA assay (*middle panel*) using different amounts of synthetic DNA input. The mutant mtDNA assay used a FAM-labeled probe, while the reference mtDNA assay used a HEX-labeled probe. The synthetic DNA input for each well is noted above the droplet plots. *Horizontal lines* are drawn to differentiate the distinct droplet populations in the mutant mtDNA assay (high-amplitude positive, low-amplitude positive, and negative) and reference mtDNA assay (positive and negative). The *bottom panel* shows the calculated percentage of each synthetic DNA. The percentage of mutant DNA input was quantified by dividing the concentration (copies/μl) of high-amplitude FAM-positive droplets by the concentration of HEX-positive droplets. The percentage of WT DNA input was quantified by dividing the concentration of low-amplitude FAM-positive droplets by the concentration of HEX-positive droplets. *C*, duplex droplet dPCR plots for the WT mtDNA assay (*top panel*) and reference mtDNA assay (*middle panel*) using different amounts of synthetic DNA input. The WT mtDNA assay used a FAM-labeled probe, while the reference mtDNA assay used a HEX-labeled probe. The synthetic DNA input for each well is noted above the droplet plots. *Horizontal lines* are drawn to differentiate the distinct droplet populations, as described above. The *bottom panel* shows the calculated percentage of each synthetic DNA input. The percentage of WT DNA input was quantified by dividing the concentration (copies/μl) of high-amplitude FAM-positive droplets by the concentration of HEX-positive droplets. The percentage of mutant DNA input was quantified by dividing the concentration of low-amplitude FAM-positive droplets by the concentration of HEX-positive droplets. NTC, no template control; mtDNA, mitochondrial DNA; dPCR, digital PCR.

To test the specificity of the mutant and WT assays, two synthetic double-strand DNA fragments were designed to contain relevant mtDNA sequence, including the amplicon surrounding the mutant and WT mtDNA assays as well as the reference mtDNA assay. One synthetic DNA fragment contained the mutant 3243G while the other contained the WT 3243A. Both were used to test the ability of the probes to specifically recognize their intended sequence and not the opposing sequence. We analyzed both synthetic DNA fragments separately using the mutant and WT mtDNA assays and found that both assays generated a high-amplitude positive population of droplets when their intended sequence was present, and a low-amplitude positive population of droplets when the opposite sequence was present (Fig. 2*A*). With either probe, both populations of droplets corresponding to the mutant and WT synthetic DNA were distinctly positive as there was a well-defined separation between the low-amplitude positive droplets and the negative droplets. Moreover, the two positive droplet populations could clearly be separated from each other based on this difference in fluorescence amplitude.

We then sought to determine if the probes could accurately quantify defined ratios of each input DNA. We duplexed both the mutant and WT mtDNA assays with a reference mtDNA assay that is present in the endogenous MT-ND2 gene and was incorporated into the designed synthetic DNA fragment. We reasoned that by using a reference assay that is the same on both templates, we could reliably quantify the total amount of input DNA and this value should equal the sum of the two positive populations found with either the mutant or WT mtDNA assays. In other words, the sum of the positive droplets corresponding to mutant DNA and the positive droplets corresponding to WT DNA should equal the number of positive reference assay droplets (mutant [FAM] + WT [FAM] = total [HEX]). We mixed the two synthetic templates in various ratios and analyzed them using a duplex of the mutant mtDNA assay and reference mtDNA assay (Fig. 2*B*) and the WT mtDNA assay and reference mtDNA assay (Fig. 2*C*).

When the mutant mtDNA assay was used, we once again detected two distinct positive droplet populations with different levels of amplitude in the FAM channel (Fig. 2*B*, top panel). When the mutant sequence was exclusively present, the fluorescence of the resulting droplet population had an amplitude of ∼5500. When the WT sequence was exclusively present, the fluorescence of the resulting droplet population had an amplitude of ∼1250. When both sequences were present, the two positive droplet populations could be separated based on this difference in fluorescence amplitude. The same was true for the WT mtDNA assay (Fig. 2*C*, top panel), although the separation between the two positive droplet populations was not as well defined. When the WT sequence was exclusively present, the resulting droplet population had a fluorescence amplitude of ∼6500. When the mutant sequence was exclusively present, the resulting droplet population had a fluorescence amplitude of ∼2500. Once again, when both sequences were present, the two positive droplet populations could be separated based on their distinct droplet amplitudes. Additionally, for both assays, we found that the sum of the concentration (copies/μl) of the two positive populations in the FAM channel did equal the concentration of reference positive droplets in the HEX channel, and the calculated frequency of each closely matched the known ratios of input DNA (Fig. 2*B*, bottom panel and 2C, bottom panel, Supplemental Tables 1 and 2). This indicates that our mutant and WT mtDNA assays are amplified as efficiently as the reference mtDNA assay, and the presence of the heteroplasmic base does not interfere with PCR efficiency. Together, these results suggest that the probes we designed are both highly sensitive for their intended target sequence and capable of discriminating and quantifying the two distinct mtDNA sequences. This finding demonstrated that only one of these assays (mutant/WT) was needed to quantify mtDNA heteroplasmy.

### dPCR can be used to quantify heteroplasmy in cellular DNA samples

**Figure 3:**
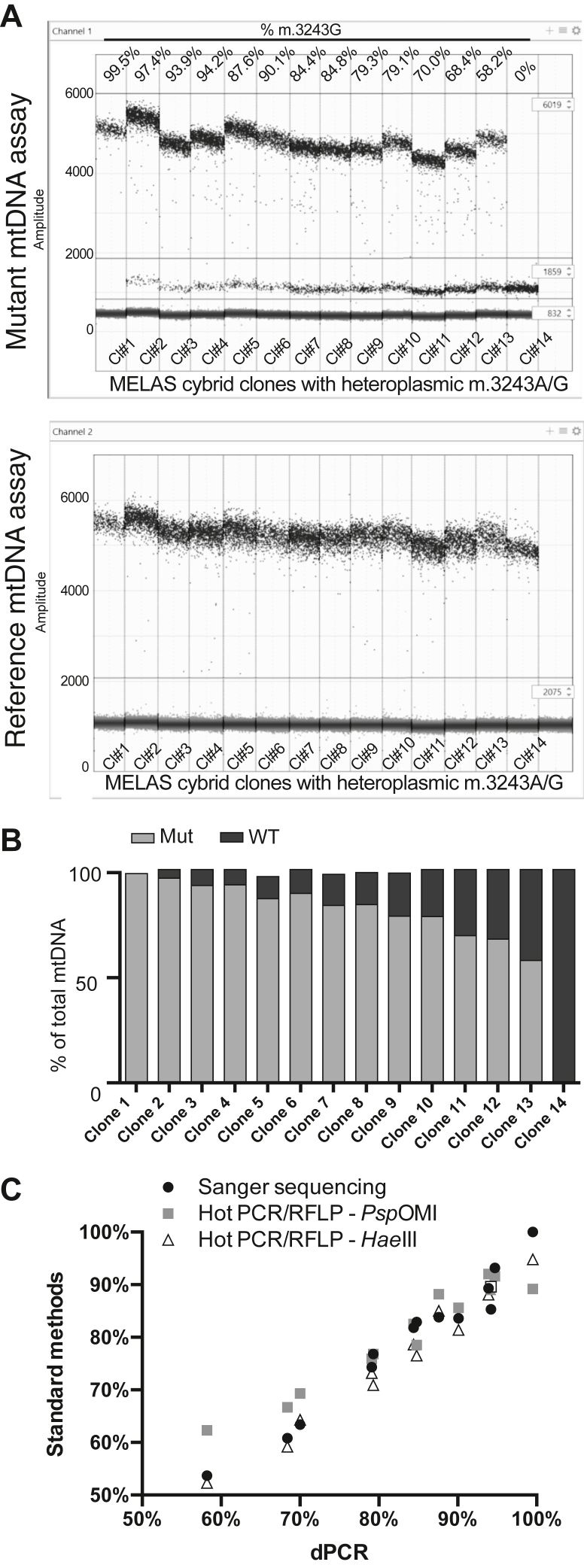
**dPCR can be used to quantify heteroplasmy in cellular DNA samples**. *A*, duplex droplet dPCR plots for the mutant mtDNA assay (*top*) and reference mtDNA assay (*bottom*) using various m.3243A > G heteroplasmic cell lines (clones #1–14). The mutant mtDNA assay used a FAM-labeled probe, while the reference mtDNA assay used a HEX-labeled probe. *Horizontal lines* are drawn to differentiate the distinct droplet populations in the mutant mtDNA assay (high-amplitude positive, low-amplitude positive, and negative) and reference mtDNA assay (positive and negative). The calculated percentage of mutant mtDNA is displayed above each sample. *B*, heteroplasmy quantification of various m.3243A > G heteroplasmic cell lines using the duplex of the mutant mtDNA assay and reference mtDNA assay shown in Fig. 3*A*. The percentage of mutant mtDNA was quantified by dividing the concentration (copies/μl) of high-amplitude FAM-positive droplets by the concentration of HEX-positive droplets. The percentage of WT mtDNA was quantified by dividing the concentration of low-amplitude FAM-positive droplets by the concentration of HEX-positive droplets. *C*, correlation analysis comparing the three standard methods for heteroplasmy quantification (Sanger sequencing, *Psp*OMI ”Last cycle hot” PCR/RFLP, and *Hae*III ”Last cycle hot” PCR/RFLP) to dPCR. The R value comparing dPCR to Sanger sequencing was 0.9938, dPCR to *Psp*OMI ”Last cycle hot” PCR/RFLP was 0.9924, and dPCR to *Hae*III ”Last cycle hot” PCR/RFLP was 0.9954. mtDNA, mitochondrial DNA; dPCR, digital PCR; RFLP, restriction fragment-length polymorphism.

A duplex of the mutant mtDNA assay and reference mtDNA assay was then used to quantify heteroplasmy levels in various m.3243A > G heteroplasmic cell lines (Fig. 3*A*). As was the case with the synthetic DNA fragments, two distinct positive droplet populations were seen in the mutant mtDNA assay (FAM channel). The percentage of mutant mtDNA was quantified by dividing the concentration (copies/μl) of the high-amplitude FAM-positive droplets by the concentration of the HEX-positive droplets. The percentage of WT mtDNA was quantified by dividing the concentration of the low-amplitude FAM-positive droplets by the concentration of the HEX-positive droplets. As was the case with the synthetic DNA fragments, the sum of the positive droplets corresponding to mutant mtDNA (high-amplitude FAM positive) and the positive droplets corresponding to WT mtDNA (low-amplitude FAM positive) consistently equaled the amount of total mtDNA detected using the reference mtDNA assay (HEX positive) (Fig. 3*B*, Supplemental Table 3).

We sought to determine how closely our dPCR heteroplasmy quantification method aligned with the current methods for mtDNA heteroplasmy determination, namely, Sanger sequencing and “last cycle hot” PCR/RFLP (7). We analyzed the same cellular DNA using both standard methods (Supplemental Fig. S1, Supplemental Table 4). “Last cycle hot” PCR/RFLP analysis was performed using two different enzymes that can differentiate the mutant site (*Psp*OMI and *Hae*III). As shown in Supplemental Fig. S1, the three different methods yielded similar mtDNA heteroplasmy quantification. When compared to the standard methods, heteroplasmy quantified by dPCR correlated strongly with each (R = 0.9938 compared to Sanger sequencing, R = 0.9924 compared to *Psp*OMI ”Last cycle hot” PCR/RFLP, R = 0.9954 compared to *Hae*III ”Last cycle hot” PCR/RFLP) (Fig. 3*C*). The strong correlation between dPCR and the current methods for mtDNA heteroplasmy quantification provides further support for the reliability of the data collected using dPCR.

### Relative quantification of mtDNA copy number using dPCR

**Figure 4:**
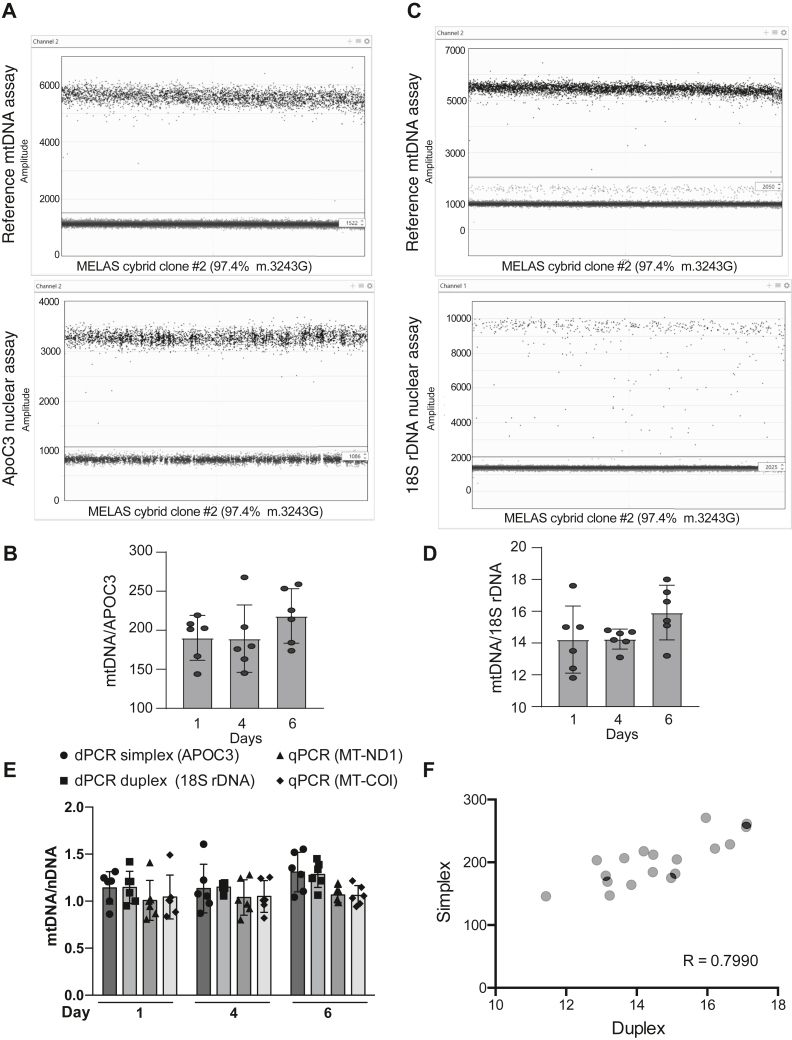
**dPCR can be used to quantify mtDNA copy number**. *A*, sample simplex droplet dPCR plots for the reference mtDNA assay (*top*) and APOC3 nuclear assay (*bottom*) using different amounts of cellular DNA isolated from clone #2. Both assays utilized HEX-labeled probes. The *solid horizontal line* indicates the threshold between negative and positive droplets for each assay. *B*, quantification of mtDNA copy number using simplex dPCR. 0.225 ng of DNA was used in the reference mtDNA assay, while 90 ng of DNA was used in the APOC3 nuclear assay. The resulting concentration (copies/μl) of positive droplets for the reference mtDNA assay was multiplied by the DNA dilution factor (400) and then divided by the concentration of positive droplets for the APOC3 nuclear assay to yield mtDNA copy number per copy of APOC3. *C*, sample duplex droplet dPCR plots for the reference mtDNA assay (*top*) and 18S rDNA nuclear assay (*bottom*) using cellular DNA isolated from clone #2. The reference mtDNA assay used a HEX-labeled probe, while the 18S rDNA nuclear assay used a FAM-labeled probe. The *solid horizontal line* indicates the threshold between negative and positive droplets for each assay. *D*, quantification of mtDNA copy number using duplex dPCR. 0.225 ng of DNA was used in the duplex containing the reference mtDNA and 18S rDNA nuclear assays. The resulting concentration (copies/μl) of positive droplets for the reference mtDNA assay was divided by the concentration of positive droplets for the 18S rDNA nuclear assay to yield mtDNA copy number per copy of 18S rDNA. *E*, comparison of mtDNA copy number by dPCR simplex, dPCR duplex, and qPCR. qPCR copy number was calculated using either MT-ND1 or MT-CO1 as the mtDNA reference gene and ACTIN as the nDNA reference gene. The calculated copy numbers were normalized to the first replicate at the first timepoint. *F*, correlation analysis of simplex dPCR and duplex dPCR for determining mtDNA copy number. The R value was 0.7990. mtDNA, mitochondrial DNA; dPCR, digital PCR; nDNA, nuclear DNA.

Another important functional determinant in mitochondrial genetics is mtDNA copy number, commonly measured as the ratio of mtDNA to nDNA. However, accurate quantitation using dPCR and Poisson distribution analysis is not precise when the number of positive droplets approaches or reaches saturation (14). Since mtDNA is present at approximately 1000 to 10,000 copies per cell (15), quantitation using a duplex of the reference mtDNA assay and a single copy nDNA gene (APOC3) was found to not be possible due to the marked difference in levels of target gene abundance in human cells (Supplemental Fig. S2*A*, Supplemental Table 5). However, a single copy gene can still be used to calculate mtDNA copy number by using a simplex dPCR approach. In this way, the DNA is diluted appropriately for the nDNA and mtDNA assays and each assay is analyzed separately, using different amounts of input DNA. The resulting concentration (copies/μl) of the more diluted sample (mtDNA) is multiplied by the dilution factor and then divided by the concentration of the less diluted sample (nDNA), thus producing the desired mtDNA/nDNA ratio. This approach was used to calculate the mtDNA copy number of a human heteroplasmic cell line (clone #2) at three separate timepoints. mtDNA copy number fluctuated between 144.0 to 267.7 mtDNA copies per copy of APOC3 over the 6 days in culture (sample droplet plots in Fig. 4*A*, quantification in Fig. 4*B*).

Alternatively, we found that a multi-copy nuclear gene could be used to duplex with the reference mtDNA assay. We chose to use 18S rDNA because the diploid human genome contains hundreds of copies of a 43-kb rDNA unit, encoding the 28, 5.8, and 18S rRNAs (16). Due to the multi-copy nature of the 18S rDNA gene, a duplex with the reference mtDNA assay was found to accurately determine copy number across multiple DNA dilutions (Supplemental Fig. S2*B*, Supplemental Table 6). The same cellular DNA samples used in Fig. 4*B* were analyzed using the duplex approach. The calculated mtDNA copy number fluctuated between 11.8 and 18.0 mtDNA copies per copy of 18S rDNA over the 6 days in culture (sample droplet plot in Fig. 4*C*, quantification in Fig. 4*D*). A distinct low-amplitude population of HEX-positive droplets appeared in the duplex that was not present when the assay was run as a simplex (Fig. 4*A*) and therefore is likely due to bleed-over from the FAM channel (17).

We compared both the simplex and duplex dPCR approaches to the current gold standard of qPCR for quantifying mtDNA copy number. We used two different mtDNA genes (MT-ND1 and MT-CO1) compared to nDNA (ACTIN) for the qPCR analysis. Normalizing all four copy number data sets to the first replicate at the first timepoint, the different methods of calculation can be compared (Fig. 4*E*). We found that dPCR, and particularly the dPCR duplex, was able to discriminate small changes in mtDNA copy number with less variability than qPCR. Despite the high degree of correlation (R = 0.7990) between the simplex and duplex dPCR approaches (Fig. 4*F*), the relative copy number data from the duplex approach eliminates human error when differentially diluting and pipetting the input DNA.

### Simultaneous quantification of mtDNA heteroplasmy and copy number using dPCR

One of the key benefits of quantifying mtDNA heteroplasmy with a single dPCR probe is the ability to duplex with other assays. In the Bio-Rad QX200 system utilized here, only two fluorescent channels can be duplexed simultaneously. From the data shown in Fig. 3, we established that the sum of the two positive droplet populations in the mutant mtDNA assay (FAM) was consistently equal to the total number of positive droplets in the reference mtDNA assay (HEX). Therefore, instead of duplexing two assays (mutant mtDNA and reference mtDNA) to determine solely heteroplasmy, we can duplex the mutant assay with an nDNA reference assay to determine heteroplasmy and mtDNA copy number simultaneously. The resulting ratio of total mtDNA (low-amplitude FAM positive + high-amplitude FAM positive) droplets to nDNA (HEX positive) droplets reflects the number of copies of mtDNA per nDNA reference gene, and the ratio of mutant mtDNA (high-amplitude FAM positive) droplets to total mtDNA (low-amplitude FAM positive + high-amplitude FAM positive) droplets can be used to simultaneously quantify heteroplasmy.

**Figure 5:**
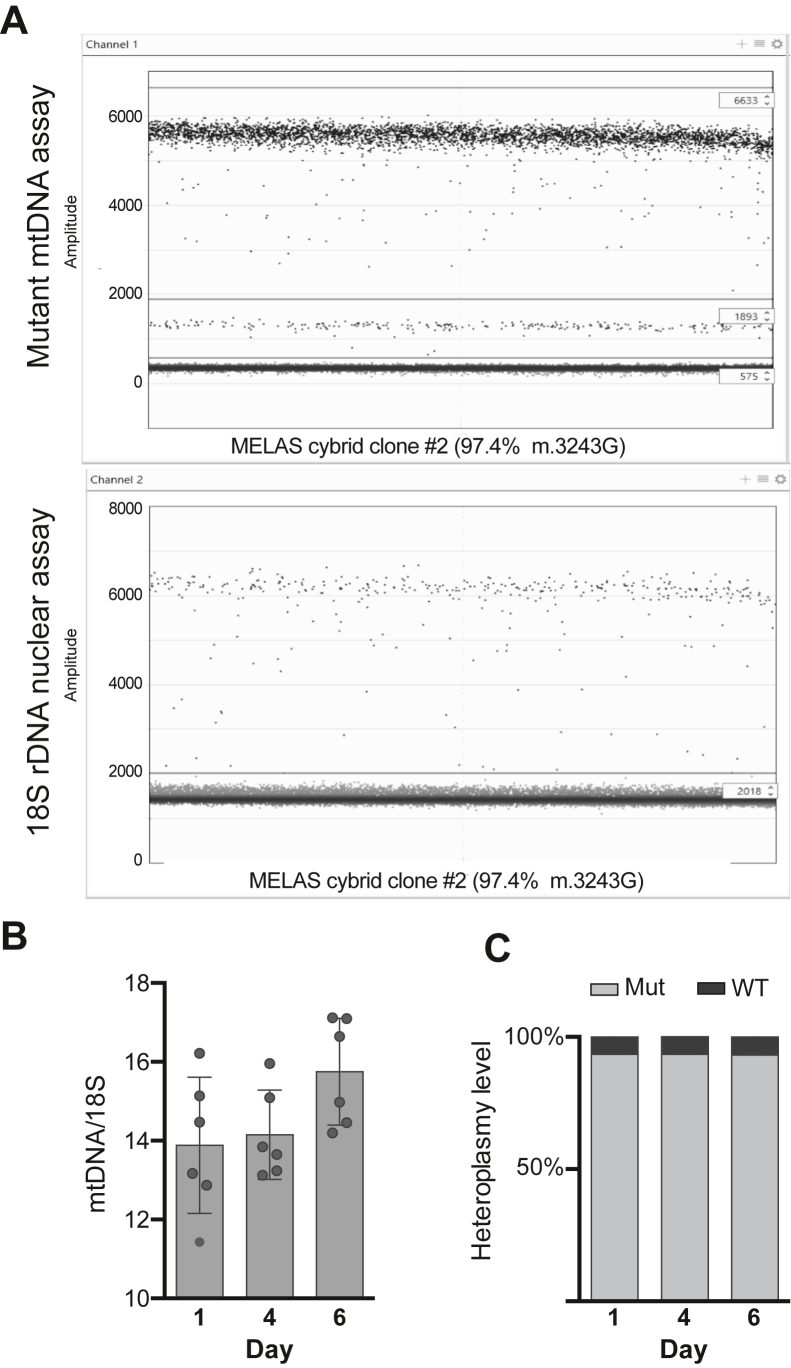
**dPCR can be used to simultaneously quantify heteroplasmy and mtDNA copy number.***A*, sample duplex droplet dPCR plots for the mutant mtDNA assay (*top*) and 18S rDNA nuclear assay (*bottom*) using cellular DNA isolated from clone #2. 0.225 ng of DNA was used in the duplex. The mutant mtDNA assay used a FAM-labeled probe, while the 18S rDNA nuclear assay used a HEX-labeled probe. *Horizontal lines* are drawn to differentiate the distinct droplet populations in the mutant mtDNA assay (high-amplitude positive, low-amplitude positive, and negative) and 18S rDNA nuclear assay (positive and negative). *B*, quantification of mtDNA copy number using duplex dPCR. mtDNA copy number was calculated by dividing the concentration (copies/μl) of both positive droplet populations (high-amplitude + low-amplitude) in the mutant mtDNA assay by the concentration of positive droplets for the 18S rDNA nuclear assay. *C*, quantification of mtDNA heteroplasmy using the mutant mtDNA assay. The percentage of mutant mtDNA was quantified by dividing the concentration (copies/μl) of high-amplitude FAM-positive droplets by the concentration of total FAM-positive droplets (high-amplitude + low-amplitude). The percentage of WT mtDNA was quantified by dividing the concentration (copies/μl) of low-amplitude FAM-positive droplets by the concentration of total FAM-positive droplets (high-amplitude + low-amplitude). mtDNA, mitochondrial DNA; dPCR, digital PCR.

A duplex of the mtDNA mutant assay and 18S rDNA nuclear assay was used to analyze the same samples described in Fig. 4. The calculated mtDNA copy number fluctuated between 11.4 and 17.1 mtDNA copies per copy of 18S rDNA over the 6 days in culture (sample droplet plot in Fig. 5*A*, quantification in Fig. 5*B*) and the heteroplasmy level in these samples was stable at 93.3% ± 0.3% (Fig. 5*C*). There was no statistically significant difference between the mtDNA copy number calculated with the duplex of the reference mtDNA assay and the 18S rDNA nuclear assay or the duplex of the mutant mtDNA assay and the 18S rDNA nuclear assay (Supplemental Fig. S3).

### General applicability of dPCR to simultaneously quantify mtDNA heteroplasmy and copy number

**Figure 6:**
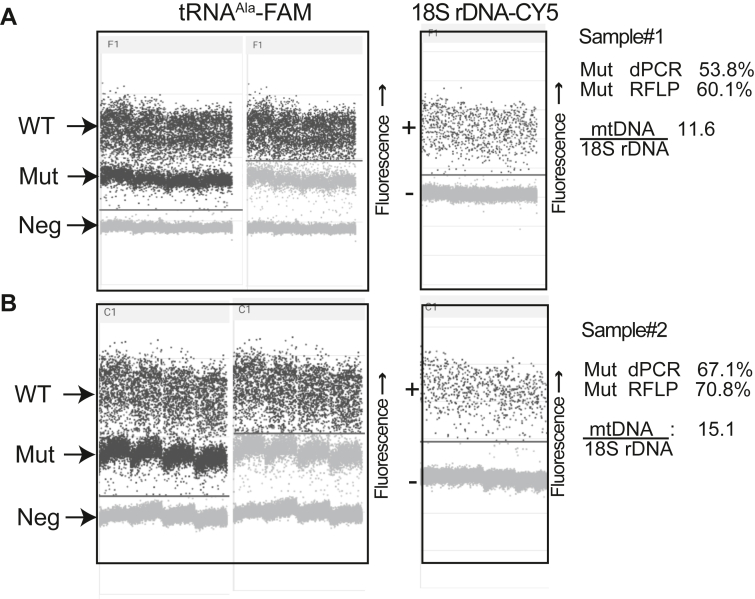
**General applicability of the dPCR approach for heteroplasmic mtDNA mutations.** dPCR was used to determine mtDNA heteroplasmy and mtDNA copy number using DNA samples isolated from heteroplasmic mice carrying the m.5024C > T mutation. DNA was isolated from the tibialis anterior (sample #1, *A*) and cortex (sample #2, *B*). Two different dPCR assays were used: one specific to the WT m.5024C and one in the mouse 18S rDNA gene. The WT assay used a FAM-labeled probe, while the 18S rDNA nuclear assay used a Cy5-labeled probe. *Horizontal lines* are drawn to differentiate the distinct droplet populations in the WT mtDNA assay (high-amplitude positive, low-amplitude positive, and negative) and 18S rDNA nuclear assay (positive and negative). The percentage of WT mtDNA was quantified by dividing the concentration (copies/μl) of high-amplitude FAM-positive droplets by the concentration of total FAM-positive droplets (high-amplitude + low-amplitude). The percentage of mutant mtDNA was quantified by dividing the concentration of low-amplitude FAM-positive droplets by the concentration of total FAM-positive droplets (high-amplitude + low-amplitude). mtDNA copy number was calculated by dividing the concentration of both positive droplet populations (high-amplitude + low-amplitude) in the WT mtDNA assay by the concentration of positive droplets for the 18S rDNA nuclear assay. The same samples used to detect the levels of heteroplasmy by dPCR were also analyzed by RFLP, as indicated on the *right*. mtDNA, mitochondrial DNA; dPCR, digital PCR; RFLP, restriction fragment-length polymorphism.

In addition to the human m.3243A > G mutation, we tested our approach on a pathogenic mouse heteroplasmic mtDNA mutation. The m.5024C > T mutation was identified in a mouse model with late onset cardiomyopathy, which is currently the only available animal model with a pathogenic mtDNA mutation (18). To test the method in a different apparatus, we used plate-based dPCR (QIAGEN) with a probe designed to preferentially bind the WT m.5024C sequence. Similar to the human m.3243G, the probe gave a higher intensity signal when bound to the WT sequence and a lower intensity signal when bound to the mutant sequence (Fig. 6, *A* and *B*, left panel). We also found that a duplex between the mouse WT mtDNA assay and mouse 18S rDNA nuclear assay (Fig. 6, *A* and *B*) allowed for simultaneous heteroplasmy and copy number quantification across different tissues.

## Discussion

Determination of mtDNA heteroplasmy and copy number are critical aspects of mitochondrial genetics, and proper quantification of both is essential to understanding mtDNA disease onset and curative measures. Very small changes in heteroplasmy can define clinical phenotypes, as has been observed for the m.3243A > G mutation (19, 20). Moreover, laboratory work requires the precise determination of mtDNA heteroplasmy and copy number during a vast array of experiments aiming to alter mtDNA heteroplasmy, including mtDNA cleavage and mtDNA base editing (15). Cell lines and mouse models are used to develop therapies for mtDNA diseases, and the monitoring of both heteroplasmy and copy number are central to these studies (21, 22, 23).

Traditionally, the determination of mtDNA heteroplasmy has been done either by Sanger sequencing, which requires additional normalization of the data to compensate for peak size differences, or by PCR/RFLP. The latter method has the drawback that late stages of a PCR reaction form a substantial number of heteroduplexes containing one strand with one haplotype paired with a strand carrying a different haplotype. Such heteroduplexes are not digested by restriction endonucleases, leading to a bias in the heteroplasmy estimation. To circumvent this problem, a “last cycle hot” PCR was developed. By introducing radioactivity in the last cycle, all labeled amplicons are homoduplexes (7). Unfortunately, this approach involves the use of radioactive material and requires a specific restriction endonuclease to recognize either the mutant or WT version of the mtDNA (24). dPCR has been used for heteroplasmy determination in the past, but this was done by using multiple allele–specific probes (12, 25, 26). The same assays used in our dPCR could be used in qPCR reactions, however qPCR mtDNA heteroplasmy quantification is influenced by template concentration and limited to cycle threshold (Ct) differences ≥ one. Moreover, qPCR multiplexing is more sensitive to amplification conditions when compared to dPCR, which has a binary output. Finally, two assays are required, one for the mutant and one for the WT, further adding potential sources of variation.

Another critical aspect of mitochondrial genetics is the determination of mtDNA copy number. This is particularly critical not only when analyzing mtDNA depletion syndromes (27) but also when mitochondrial-targeted nucleases are used to eliminate mutant mtDNA (15, 28). qPCR has been the traditional method for mtDNA copy number determinations, but small changes (less than 2-fold) cannot be accurately determined by changes in Ct (11).

The dPCR method described here is unique as it allows for precise and simultaneous determination of mtDNA heteroplasmy and copy number in a duplex assay, with mutant and WT mtDNA being detected and discriminated by a singular primer/probe set. The data generated is precise, robust, and does not require standard curves. Errors in pipetting are minimized by the duplex nature of the assay. Amplification of nuclear mitochondrial pseudogenes (29) is inherently avoided due to the massive sample dilution that is necessary to amplify mtDNA without reaching saturation of dPCR positive droplets (Supplemental Fig. S2*A*). Nonetheless, caution should be exercised when detecting extremely low levels of heteroplasmy. Besides the QX200 droplet dPCR platform (Bio-Rad), we have also used the QIAcuity (QIAGEN), which uses physical compartments instead of droplets. Both methods were highly efficient and sensitive for the determination of mtDNA heteroplasmy and copy number. The use of recently developed chemistries that increase the probe T_M_, including proprietary modifications, as used in this study, as well as locked nucleic acids (30) and minor grove binding (31) containing probes help to improve probe specificity and allow for the discrimination against a single base pair.

Several limitations to the method described here should be noted. First, drawbacks of probe-based assays also apply to dPCR, including DNA regions with specific secondary structures or unbalanced G:C content that may be difficult to bind. Additionally, the dPCR method described here is only useful for quantifying known mutations, not identifying *de novo* ones (32). Finally, proper dilution of input DNA is imperative to generate data that falls within a Poisson distribution. If there are not enough empty droplets, Poisson statistics cannot accurately be applied (33) (https://www.bio-rad.com/webroot/web/pdf/lsr/literature/Bulletin_6407.pdf).

As gene editing in mtDNA becomes a reality (15, 28), a quick and relatively inexpensive method, such as the dPCR duplex approach described here, would be able to scan isolated DNA for predicted changes. Beyond mitochondrial genetics, this approach can also be adapted for somatic mutations. For example, specific tumor-related SNPs could be tested in samples that contain low levels of cancer cells (34, 35). Even though cancer research has taken full advantage of dPCR technology to search for rare events (36), the simultaneous detection of WT and mutant alleles in a mixed population has several advantages.

## Experimental procedures

### Design of human dPCR assays

To generate dPCR assays specific to the mutant 3243G and WT 3243A mtDNA sequences, two BHQplus probes (Biosearch Technologies) were designed using RealTimeDesign software (Biosearch Technologies) with a target T_M_ of 68 ºC. The probes were designed using the SNP genotyping application, with base specificity on the 3243 position, specifically to either the mutant (G) or WT (A) haplotype. Primers surrounding the probes were designed using PrimerQuest tool (Integrated DNA Technologies) with a target T_M_ of 62 ºC, amplicon length <150 bp, primer concentration of 900 nM, probe concentration of 250 nM, and magnesium concentration of 3.8 mM. The same primers were used for both assays. A reference mtDNA assay downstream of the heteroplasmic base was designed using PrimerQuest tool (Integrated DNA Technologies) with the same primer and probe target T_M_ and amplicon size. nDNA reference assays were designed in the same manner in the nuclear genes of APOC3 and 18S rDNA.

### Design of synthetic double-strand DNA fragments

The mtDNA sequence surrounding both the mutant/WT mtDNA assays and the reference mtDNA assay were placed into a synthetic fragment totaling 735 base pairs (Integrated DNA Technologies). Two synthetic fragments were designed: one containing the mutant 3243G base and one containing the WT 3243A base.

### Cloning and linearization of plasmid DNA

The two synthetic fragments described above were cloned into a Zero Blunt cloning vector (Thermo Fisher Scientific). One microgram of each plasmid was then linearized with *Sac*I HF (New England Biolabs), which is found in the plasmid backbone and gel purified using NucleoSpin Gel and PCR Clean-up Columns (Macherey-Nagel).

### dPCR on linearized plasmids

Droplet dPCR was conducted using the mutant mtDNA, WT mtDNA, and reference mtDNA assays indicated in Supplemental Table 7. dPCR amplifications were duplexed in a 24 μl reaction containing 1× dPCR Supermix for Probes (no dUTP; Bio-Rad), 250 nM of each probe, 900 nM of each primer, and 90 fg of linearized plasmid DNA. Droplets were generated using a QX200 droplet generator (Bio-Rad), and PCR was performed on a C1000 Touch thermal cycler (Bio-Rad). Cycling conditions were as follows: one cycle of 95 ºC (2 ºC/s ramp) for 10 min, 45 cycles of 94 ºC (2 ºC/s ramp) for 10 s, 59.2 ºC (2 ºC/s ramp) for 30 s, 72 ºC (0.2 ºC/s ramp) for 1 min, one cycle of 98 ºC for 10 min, four ºC hold. Droplets were analyzed using a QX200 droplet reader (Bio-Rad), and QuantaSoft analysis software (Bio-Rad) was used to acquire and analyze data.

### Cell culture and reagents

Cybrid cells were derived from the fusion of 143B osteosarcoma cells (ATCC CRL-8303) depleted of mtDNA (37) with mitochondrial encephalomyopathy, lactic acidosis, and stroke-like episodes m.3243A > G patient fibroblasts. Cells were maintained in Dulbecco’s Modified Eagle Medium (Thermo Fisher Scientific) with 10% fetal bovine serum (Thermo Fisher Scientific), 1 mM sodium pyruvate (Thermo Fisher Scientific), 50 μg/ml uridine (MilliporeSigma), 20 μg/ml gentamycin (Thermo Fisher Scientific), and 5 μg/ml Plasmocin (InvivoGen) and grown in 5% CO_2_, 37 ºC humidity-controlled incubators.

### Cellular DNA isolation

DNA was isolated from cultured cells using the NucleoSpin Blood QuickPure kit (Macherey-Nagel). Cellular DNA concentrations were obtained using the Lunatic UV/Vis absorbance spectrometer (Unchained Labs).

### ”Last cycle hot” PCR/RFLP analysis

PCR amplicons were obtained using the primers indicated in Supplemental Table 8. Amplicons were generated in a reaction containing 1× DreamTaq green buffer (Thermo Fisher Scientific), 50 nmol of each primer, 2.5 μmol of dNTPs, 1U of DreamTaq polymerase (Thermo Fisher) in a final volume of 15 μl. Cycling conditions were as follows: one cycle of 94 ºC for 5 min, 30 cycles of 94 ºC for 1 min, 59 ºC for 1 min, 72 ºC for 30 s, one cycle of 72 ºC for 5 min, 12 ºC hold. Resulting PCR products were subjected to one additional cycle with 32PdCTP. Radiolabeled products are expected to be homoduplexes as they are not denatured after the extension step (22). Labeled products were digested with 0.5 μl *Psp*OMI (10U) or *Hae*III (0.5U) (New England Biolabs) at 37 ºC for 3 h. *Psp*OMI generates two fragments (178 and 176 base pairs) when mutant mtDNA is present, as opposed to the singular WT (354 base pairs) fragment. *Hae*III generates four fragments (97, 97, 81, and 72 base pairs) when the mutant sequence is present and three fragments (169, 97, and 81 base pairs) when the WT sequence is present. After digestion, products were run in a 12% PAGE, and signal was detected and quantified using a Cyclone Phosphoimager (PerkinElmer).

### Sanger sequencing analysis

The same PCR fragments described for PCR/”Last cycle hot” RFLP analysis were analyzed by Genewiz (South Plainfield) Sanger sequencing using the forward primer for mtDNA heteroplasmy described in Supplemental Table 8. Peaks were analyzed by QSVAnalyzer for peak ratios (38).

### m.3243A > G heteroplasmy dPCR

Droplet dPCR was conducted using the mutant mtDNA and reference mtDNA assays indicated in Supplemental Table 7. dPCR amplifications were duplexed in a 24 μl reaction containing 1× dPCR Supermix for Probes (no dUTP; Bio-Rad), 250 nM of each probe, 900 nM of each primer, 20U/μl Hind-III HF, and 0.225 ng cellular DNA. Droplets were generated using a QX200 droplet generator (Bio-Rad), and PCR was performed on a C1000 Touch thermal cycler (Bio-Rad). Cycling conditions were as follows: one cycle of 95 ºC (2 ºC/s ramp) for 10 min, 45 cycles of 94 ºC (2 ºC/s ramp) for 10 s, 59.2 ºC (2 ºC/s ramp) for 30 s, 72 ºC (0.2 ºC/s ramp) for 1 min, one cycle of 98 ºC for 10 min, four ºC hold. Droplets were analyzed using a QX200 droplet reader (Bio-Rad), and QuantaSoft analysis software (Bio-Rad) was used to acquire and analyze data. The percentage of mutant mtDNA was calculated by dividing the concentration (copies/μl) of the mutant mtDNA (high-amplitude FAM positive) droplets by the concentration of the total mtDNA (HEX positive) droplets. The percentage of WT mtDNA was calculated by dividing the concentration of the WT mtDNA (low-amplitude FAM positive) droplets by the concentration of the total mtDNA (HEX positive) droplets.

### Copy number dPCR–simplex (single copy nuclear gene - APOC3)

Droplet dPCR was conducted using the reference mtDNA and APOC3 nDNA assays indicated in Supplemental Table 7. dPCR amplifications were analyzed as a simplex in a 24 μl reaction containing 1× dPCR Supermix for Probes (no dUTP; Bio-Rad), 250 nM of probe, 900 nM of each primer, 20U/μl Hind-III HF, and cellular DNA. For the APOC3 nuclear assay, 90 ng cellular DNA was added. For the reference mtDNA assay, 0.225 ng cellular DNA was added. Droplets were generated using a QX200 droplet generator (Bio-Rad), and PCR was performed on a C1000 Touch thermal cycler (Bio-Rad). Cycling conditions were as follows: one cycle of 95 ºC (2 ºC/s ramp) for 10 min, 45 cycles of 94 ºC (2 ºC/s ramp) for 10 s, 59.2 ºC (2 ºC/s ramp) for 30 s, 72 ºC (0.2 ºC/s ramp) for 1 min, one cycle of 98 ºC for 10 min, four ºC hold. Droplets were analyzed using a QX200 droplet reader (Bio-Rad), and QuantaSoft analysis software (Bio-Rad) was used to acquire and analyze data. mtDNA copy number was calculated by multiplying the concentration (copies/μl) of reference mtDNA–positive droplets by the dilution factor (400) and dividing by the concentration of APOC3-positive droplets.

### Copy number dPCR–duplex (multi-copy nuclear gene - 18S rDNA)

Droplet dPCR was conducted using the reference mtDNA and 18S rDNA reference assays indicated in Supplemental Table 7. dPCR amplifications were duplexed in a 24 μl reaction containing 1× dPCR Supermix for Probes (no dUTP; Bio-Rad), 250 nM of each probe, 900 nM of each primer, 20U/μl Hind-III HF, and 0.225 ng cellular DNA. Droplets were generated using a QX200 droplet generator (Bio-Rad), and PCR was performed on a C1000 Touch thermal cycler (Bio-Rad). Cycling conditions were as follows: one cycle of 95 ºC (2 ºC/s ramp) for 10 min, 45 cycles of 94 ºC (2 ºC/s ramp) for 10 s, 59.2 ºC (2 ºC/s ramp) for 30 s, 72 ºC (0.2 ºC/s ramp) for 1 min, one cycle of 98 ºC for 10 min, four ºC hold. Droplets were analyzed using a QX200 droplet reader (Bio-Rad), and QuantaSoft analysis software (Bio-Rad) was used to acquire and analyze data. mtDNA copy number was calculated by dividing the concentration (copies/μl) of reference mtDNA (HEX positive) droplets by the concentration of 18S rDNA (FAM positive) droplets.

### Simultaneous evaluation of m.3243A > G heteroplasmy and copy number by dPCR

Droplet dPCR was conducted using the mutant mtDNA and 18S rDNA reference assays indicated in Supplemental Table 7. dPCR amplifications were duplexed in a 24 μl reaction containing 1× dPCR Supermix for Probes (no dUTP; Bio-Rad), 250 nM of each probe, 900 nM of each primer, 20U/μl Hind-III HF, and 0.225 ng cellular DNA. Droplets were generated using a QX200 droplet generator (Bio-Rad), and PCR was performed on a C1000 Touch thermal cycler (Bio-Rad). Cycling conditions were as follows: one cycle of 95 ºC (2 ºC/s ramp) for 10 min, 45 cycles of 94 ºC (2 ºC/s ramp) for 10 s, 59.2 ºC (2 ºC/s ramp) for 30 s, 72 ºC (0.2 ºC/s ramp) for 1 min, one cycle of 98 ºC for 10 min, four ºC hold. Droplets were analyzed using a QX200 droplet reader (Bio-Rad), and QuantaSoft analysis software (Bio-Rad) was used to acquire and analyze data. The percentage of mutant mtDNA was calculated by dividing the concentration (copies/μl) of the mutant mtDNA (high-amplitude FAM positive) droplets by the concentration of the total mtDNA (low-amplitude FAM positive + high-amplitude FAM positive) droplets. The percentage of WT mtDNA was calculated by dividing the concentration of the WT mtDNA (low-amplitude FAM positive) droplets by the concentration of the total mtDNA (low-amplitude FAM positive + high-amplitude FAM positive) droplets. mtDNA copy number was calculated by dividing the concentration of total mtDNA (low-amplitude FAM positive + high-amplitude FAM positive) droplets by the concentration of nDNA (HEX positive) droplets.

### Copy number qPCR

qPCR was performed using PrimeTime qPCR Assay (Integrated DNA Technologies) and the primers/probes indicated in Supplemental Table 9. PCR was performed on a CFX96/C1000 qPCR machine (Bio-Rad). Cycling conditions were as follows: one cycle of 95 ºC for 3 min, 39 cycles of 95 ºC for 15 s, 60 ºC for 1 min. Comparative Ct method was used to determine relative reads. Total mtDNA levels were determined by comparing mtDNA MT-ND1 or MT-COX1 to nDNA ACTIN.

### Design of mouse dPCR assays

To generate dPCR assays specific to the mouse WT 5024C mtDNA sequences, a BHQplus probe (Biosearch Technologies) was designed using RealTimeDesign software (Biosearch Technologies) with a target T_M_ of 68 ºC. The probe was designed using the SNP genotyping application, with base specificity on the 5024 position, specifically to the WT (C) haplotype. Primers surrounding the probes were designed using PrimerQuest tool (Integrated DNA Technologies) with a target T_M_ of 62 ºC, amplicon length <150 bp, primer concentration of 900 nM, probe concentration of 250 nM, and magnesium concentration of 3.8 mM. An nDNA reference assay in the 18S rDNA gene was designed using PrimerQuest tool (Integrated DNA Technologies) with the same primer and probe target T_M_ and amplicon size.

### Simultaneous evaluation of m.5024C > T heteroplasmy and copy number by dPCR

dPCR with the QIAcuity (QIAGEN) was conducted using the mouse WT mtDNA assay and the mouse 18S rDNA nuclear assay indicated in Supplemental Table 10. dPCR was performed in 24 well/nanoplates 26K (QIAGEN) with 40 μl total reaction volume containing 1× QIAcuity probe master mix (QIAGEN), 0.4 μM of probe, 1.6 μM of each primer, 0.25U/μl EcoRI-HF, and 0.9 ng cellular DNA. Cycling conditions were as follows: one cycle of 95 ºC for 2 min, 40 cycles of 95 ºC for 15 s, 57 ºC for 1 min. Results were analyzed with the QIAcuity Software Suite (QIAGEN).

### Statistical analysis

All data analysis was performed using GraphPad Prism 9. Correlation was measured using Pearson’s correlation coefficient (R). Pairwise comparisons were performed using the unpaired two-tailed Student’s *t* test. *p*-values of ≤0.05 were considered significant.

## Data availability

Data available within the article or its supplementary materials. Any additional details regarding the data that support the findings of this study are available on request from the corresponding authors [W. K. S. or C. T. M.].

## Supporting information

This article contains supporting information.

## Conflict of interest

Wendy Shoop and Cassandra Gorsuch are employees and conducted these experiments at Precision BioSciences. Sandra Bacman and Carlos T. Moraes collaborate with Precision BioSciences scientists.
