# Supplementary material for: Precise and simultaneous quantification of mitochondrial DNA heteroplasmy and copy number by digital PCR
Source: J Biol Chem. 2022 Oct 6;298(11):102574. doi: 10.1016/j.jbc.2022.102574 (PMC9650046; doi:10.1016/j.jbc.2022.102574)
Supplement: Supplemental Tables [file mmc1.pdf]

Supplemental table 1 – Amplification of defined templates using the mutant mtDNA assay

Synthetic DNA input	Percentage of mutant measured	Percentage of WT measured
100% mutant	97.22%	0.15%
83% mutant, 17% WT	80.69%	16.44%
66% mutant, 33% WT	62.21%	35.20%
50% mutant, 50% WT	45.78%	52.42%
33% mutant, 66% WT	31.86%	65.57%
17% mutant, 83% WT	17.06%	80.98%
100% WT	0.09%	97.90%

Supplemental table 2 – Amplification of defined templates using the WT mtDNA assay

Synthetic DNA input	Percentage of mutant measured	Percentage of WT measured
100% mutant	97.57%	0.09%
83% mutant, 17% WT	78.37%	18.96%
66% mutant, 33% WT	64.81%	33.90%
50% mutant, 50% WT	48.83%	50.34%
33% mutant, 66% WT	34.01%	63.71%
17% mutant, 83% WT	14.31%	85.40%
100% WT	0.82%	97.12%

Supplemental table 3 – Heteroplasmy quantification of m.3243A>G heteroplasmic cell lines by droplet dPCR

Clone	Percentage of mutant mtDNA	Percentage of WT mtDNA
1	99.5%	0%
2	97.4%	4.7%
3	93.9%	6.7%
4	94.2%	7.1%
5	87.6%	10.5%
6	90.1%	11.9%
7	84.4%	14.8%
8	84.8%	15.2%
9	79.3%	20.5%
10	79.1%	23.4%
11	70.0%	30.1%
12	68.4%	32.3%
13	58.2%	42.7%
14	0%	103.5%

Supplemental table 4 – Percentage of mutant mtDNA quantified by various methods

**Clone**	**Sanger sequencing**	***Psp*OMI ”Last cycle hot” PCR/RFLP**	***Hae*III ”Last cycle hot” PCR/RFLP**
1	100.0%	89.2%	94.8%
2	93.2%	91.6%	91.6%
3	89.3%	92.0%	88.1%
4	85.3%	89.6%	89.0%
5	83.8%	88.2%	85.0%
6	83.6%	85.6%	81.4%
7	81.8%	82.5%	78.6%
8	82.9%	78.5%	76.5%
9	76.8%	76.8%	70.9%
10	74.3%	75.9%	73.2%
11	63.4%	69.3%	64.3%
12	60.8%	66.7%	59.2%
13	53.7%	62.3%	52.3%
14	0%	0%	0%

Supplemental table 5 – Duplex dPCR concentrations (copies/µL) of the APOC3 nuclear assay and reference mtDNA assay using various amounts of DNA input

Input DNA (ng)	Concentration (copies/µL) APOC3 nuclear assay	Concentration (copies/µL) reference mtDNA assay
90.00	427	11,608
9.00	39.4	7,753
0.90	3.9	1,159
0.45	2.04	639
0.23	1.19	330
0.11	0.236	159
0.09	0.618	148
NTC	0	0

Supplemental table 6 – Duplex dPCR concentrations (copies/µL) of the 18S rDNA nuclear assay and reference mtDNA assay using various amounts of DNA input

Input DNA (ng)	Concentration (copies/µL) 18S rDNA nuclear assay	Concentration (copies/µL) reference mtDNA assay
90.00	10,192	1,000,000
9.00	945	10,350
0.90	111	1,469
0.45	54.9	733
0.23	30.4	385
0.11	14.1	201
0.09	13.4	158
NTC	0.142	0

Supplemental table 7 – Human dPCR primers/probes

Assay	Primer/probe	Sequence (5’-3’)
Mutant mtDNA	Probe (BHQPlus)	/FAM/TGGCAGGGCCCGGT/BHQplus/
	Forward primer	CCCAAGAACAGGGTTTGTTAAG
	Reverse primer	GGAATGCCATTGCGATTAG
WT mtDNA	Probe (BHQPlus)	/FAM/ACCGGGCTCTGCCAT/BHQplus/
	Forward primer	CCCAAGAACAGGGTTTGTTAAG
	Reverse primer	GGAATGCCATTGCGATTAG
Reference mtDNA – MT-ND2	Probe	/5HEX/AGCAGTTCT/ZEN/ACCGTACAACCCTAACA/3IABkFQ/
	Forward primer	GGCAGTTGAGGTGGATTA
	Reverse primer	GGAATGCGGTAGTAGTTAGG
nDNA reference – APOC3	Probe	/5HEX/CCAGCAGGC/ZEN/CAGGTACACC/3IABkFQ/ OR /56-FAM/ CCAGCAGGC/ZEN/CAGGTACACC/3IABkFQ/
	Forward primer	ACCGCCAAGGATGCAC
	Reverse primer	GCGGGTGGGAATGGAG
nDNA reference – 18S rDNA	Probe	/5HEX/AACCAGACA/ZEN/AATCGCTCCACCAAC/3IABkFQ/ OR /56-FAM/ AACCAGACA/ZEN/AATCGCTCCACCAAC/3IABkFQ/
	Forward primer	CGGACAGGATTGACAGATT
	Reverse primer	CCAGAGTCTCGTTCGTTATC

Supplemental table 8 – Primers for PCR/"Last cycle hot" RFLP and Sanger sequencing

Assay	Primer	Sequence (5'-3')
mtDNA heteroplasmy	Forward primer	TGAGTTCAGACCGGAGTAATCCAG
	Reverse primer	GTTGGGGCCTTTGCGTAGTTGTAT

Supplemental table 9 – qPCR primers/probes

Assay	Primer/probe	Sequence (5'-3')
mtDNA reference – MT-ND1	Probe	/5TET/AAGGGTGGA/ZEN/GAGGTTAAAGGAGCC/3IABkFQ/
	Forward primer	GAAGTCACCCTAGCCATCATTC
	Reverse primer	GCAGGAGTAATCAGAGGTGTTC
mtDNA reference – MT-CO1	Probe	/56-FAM/TCCTACTCC/ZEN/TGCTCGCATCTGCTA/3IABkFQ/
	Forward primer	TTCTGACTCTTACCTCCCTCTC
	Reverse primer	TGGGAGTAGTTCCCTGCTAA
nDNA reference – ACTIN	Probe	/5Cy5/TGCCAGTGGTACGGCCAGAG/3IAbRQSp/
	Forward primer	GTCACCGGAGTCCATCAC
	Reverse primer	GCCATGTACGTTGCTATCCA

Supplemental table 10 – Mouse dPCR primers/probes

Assay	Primer/probe	Sequence (5'-3')
WT mtDNA	Probe (BHQPlus)	/FAM/AACTTCTGATAAGGACTGTAAGAC/BHQplus/
	Forward primer	GCCTTCAAAGCCCTAAGA
	Reverse primer	CGGCGGTAGAAGTAGATTG
nDNA reference - 18S rDNA	Probe	/5Cy5/AGAAACGGC/TAO/TACCACATCCAAGGA/3IAbRQSp/
	Forward primer	CGTCTGCCCTATCAACTTT
	Reverse primer	CCTCGAAAGAGTCCTGTATTG
