# Supplementary material for: Precise and simultaneous quantification of mitochondrial DNA heteroplasmy and copy number by digital PCR
Source: J Biol Chem. 2022 Oct 6;298(11):102574. doi: 10.1016/j.jbc.2022.102574 (PMC9650046; doi:10.1016/j.jbc.2022.102574)
Supplement: Supplemental Figures [file mmc2.pptx]

## Slide 1
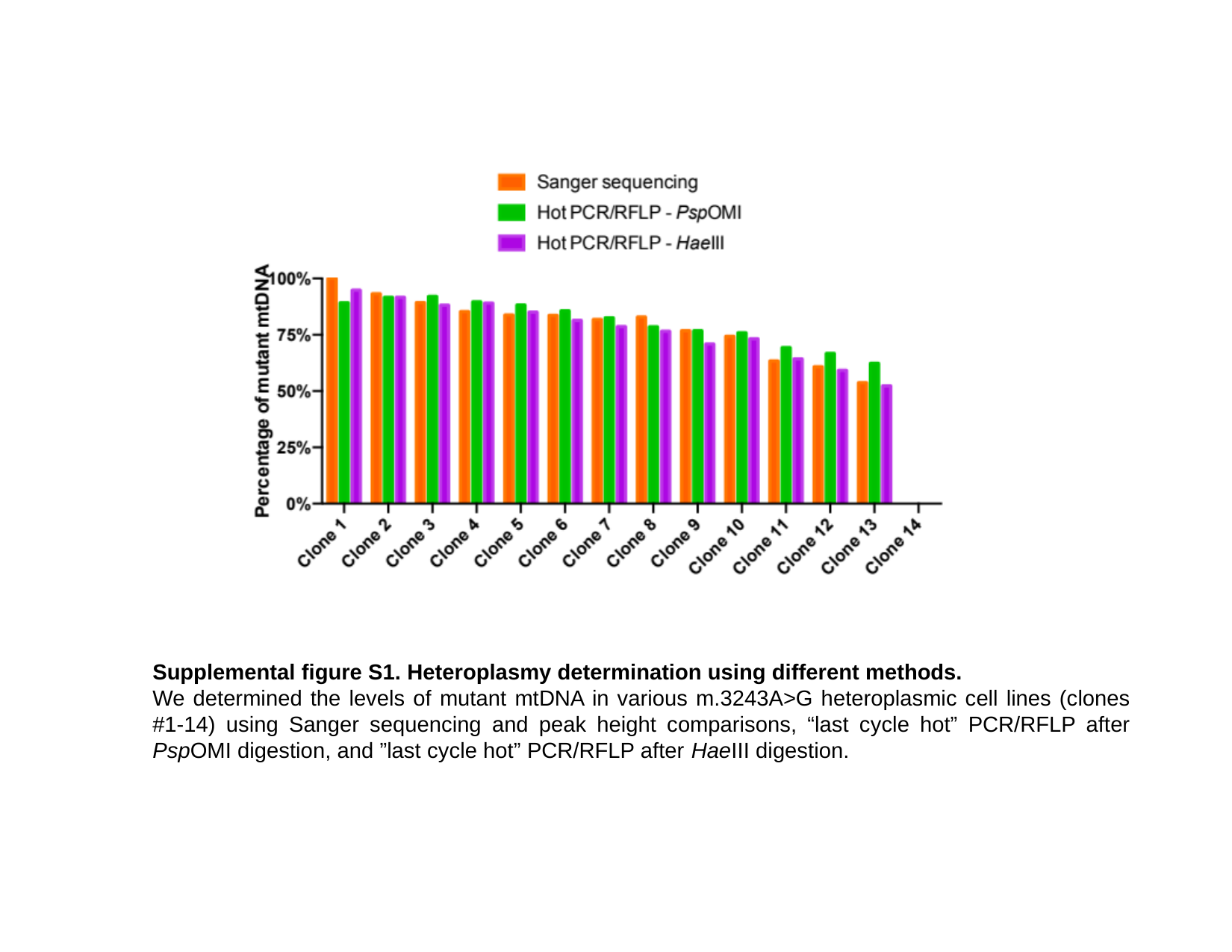

Supplemental figure S1. Heteroplasmy determination using different methods.
We determined the levels of mutant mtDNA in various m.3243A>G heteroplasmic cell lines (clones #1-14) using Sanger sequencing and peak height comparisons, “last cycle hot” PCR/RFLP after PspOMI digestion, and ”last cycle hot” PCR/RFLP after HaeIII digestion.

## Slide 2
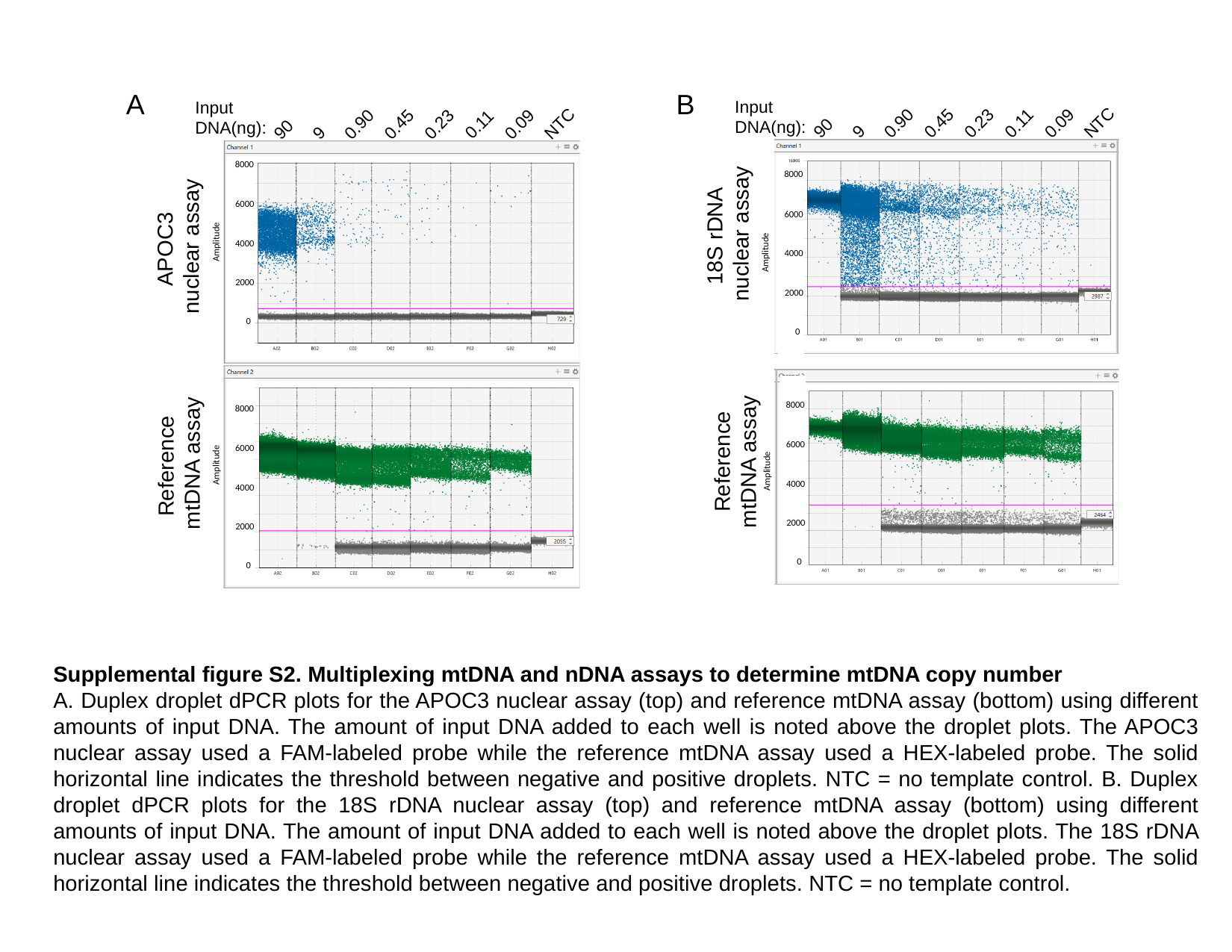

A
B
Input
DNA(ng):
Input
DNA(ng):
NTC
0.90
0.45
0.23
0.11
0.09
NTC
0.90
0.45
0.23
0.11
0.09
90
90
9
9
8000
8000
6000
18S rDNA
nuclear assay
6000
APOC3
nuclear assay
Amplitude
4000
Amplitude
4000
2000
2000
0
0
8000
8000
Reference
mtDNA assay
Reference
mtDNA assay
6000
6000
Amplitude
Amplitude
4000
4000
2000
2000
0
0
Supplemental figure S2. Multiplexing mtDNA and nDNA assays to determine mtDNA copy number
A. Duplex droplet dPCR plots for the APOC3 nuclear assay (top) and reference mtDNA assay (bottom) using different amounts of input DNA. The amount of input DNA added to each well is noted above the droplet plots. The APOC3 nuclear assay used a FAM-labeled probe while the reference mtDNA assay used a HEX-labeled probe. The solid horizontal line indicates the threshold between negative and positive droplets. NTC = no template control. B. Duplex droplet dPCR plots for the 18S rDNA nuclear assay (top) and reference mtDNA assay (bottom) using different amounts of input DNA. The amount of input DNA added to each well is noted above the droplet plots. The 18S rDNA nuclear assay used a FAM-labeled probe while the reference mtDNA assay used a HEX-labeled probe. The solid horizontal line indicates the threshold between negative and positive droplets. NTC = no template control.

## Slide 3
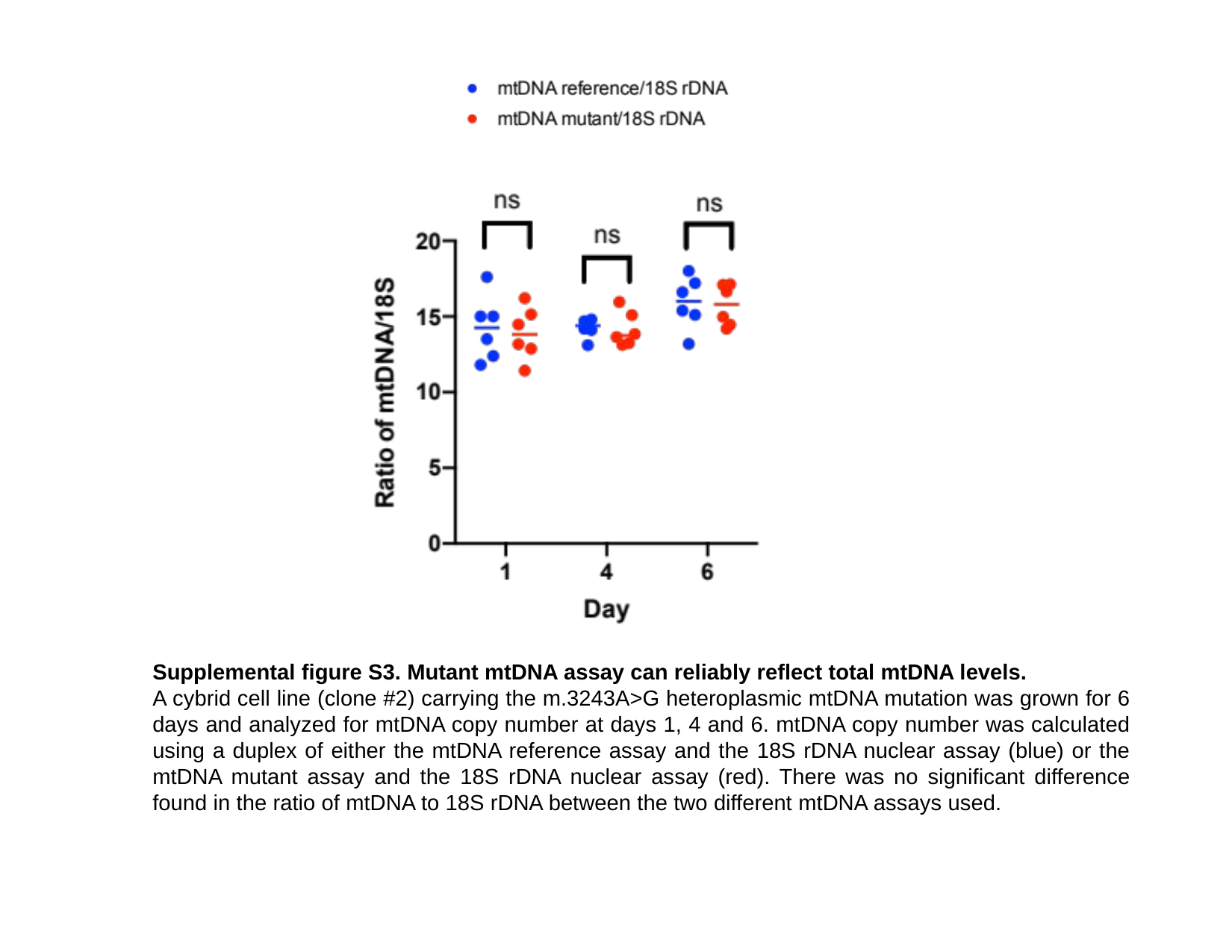

Supplemental figure S3. Mutant mtDNA assay can reliably reflect total mtDNA levels.
A cybrid cell line (clone #2) carrying the m.3243A>G heteroplasmic mtDNA mutation was grown for 6 days and analyzed for mtDNA copy number at days 1, 4 and 6. mtDNA copy number was calculated using a duplex of either the mtDNA reference assay and the 18S rDNA nuclear assay (blue) or the mtDNA mutant assay and the 18S rDNA nuclear assay (red). There was no significant difference found in the ratio of mtDNA to 18S rDNA between the two different mtDNA assays used.
